# Autophagy and Liver Ischemia-Reperfusion Injury

**DOI:** 10.1155/2015/417590

**Published:** 2015-03-15

**Authors:** Raffaele Cursio, Pascal Colosetti, Jean Gugenheim

**Affiliations:** ^1^Service de Chirurgie Digestive et Transplantation Hépatique, Hôpital l'Archet 2, Université de Nice Sophia Antipolis, 151 route Saint Antoine de Ginestière, 06200 Nice Cedex 3, France; ^2^Inserm U1065-C3M, Equipe 2, Université de Nice Sophia Antipolis, 06204 Nice Cedex 3, France

## Abstract

Liver ischemia-reperfusion (I-R) injury occurs during liver resection, liver transplantation, and hemorrhagic shock. The main mode of liver cell death after warm and/or cold liver I-R is necrosis, but other modes of cell death, as apoptosis and autophagy, are also involved. Autophagy is an intracellular self-digesting pathway responsible for removal of long-lived proteins, damaged organelles, and malformed proteins during biosynthesis by lysosomes. Autophagy is found in normal and diseased liver. Although depending on the type of ischemia, warm and/or cold, the dynamic process of liver I-R results mainly in adenosine triphosphate depletion and in production of reactive oxygen species (ROS), leads to both, a local ischemic insult and an acute inflammatory-mediated reperfusion injury, and results finally in cell death. This process can induce liver dysfunction and can increase patient morbidity and mortality after liver surgery and hemorrhagic shock. Whether autophagy protects from or promotes liver injury following warm and/or cold I-R remains to be elucidated. The present review aims to summarize the current knowledge in liver I-R injury focusing on both the beneficial and the detrimental effects of liver autophagy following warm and/or cold liver I-R.

## 1. Introduction

Partial or complete interruption of the liver blood flow and, consequently, interruption of its oxygen supply, followed by reperfusion and reestablishment of blood flow and oxygen supply, characterizes the liver ischemia-reperfusion (I-R) process. The cellular injuries caused by the ischemic period are aggravated by reperfusion [1–4]. Not only liver transplantation or resection but also liver injury following blunt or penetrating abdominal trauma [5] and hemorrhagic shock [6] may cause low liver blood flow resulting in insufficient perfusion. Finally after reperfusion liver I-R injury occurs. Liver I-R injury following hemorrhagic shock remains a major cause of morbidity and mortality after trauma [6]. Liver I-R rapidly leads to an acute inflammatory response, causing significant hepatocellular damage and organ dysfunction. The severity of injury ranges from moderately raised levels of serum aminotransferases to posthepatectomy insufficiency after liver resection or to primary nonfunction or initial poor graft function after liver transplantation [7–13]. Under extreme circumstances, multiple organ failure and death may occur. More precisely, in liver transplantation, up to 10% of early transplant failures are caused by I-R injury with a higher incidence of both acute and chronic graft rejection increasing the need for retransplantation [9–13]. Liver I-R may also be responsible for ischemic-type biliary lesions (ITBls) [14–16] and late allograft failure [17, 18]. The use of marginal liver grafts from non-heart-beating donors, older and/or steatotic organs, and of organs that have been subjected to prolonged periods of warm ischemia and cold storage, has increased in the last years due to organ shortage [19]. These grafts are more vulnerable to warm/cold I-R injury [19] underlining the need of therapeutic strategies to reduce liver I-R injury in order to improve graft viability [20].

Necrosis represents the main mode of liver cell death following warm/cold I-R [21–23], but other modes of cell death, namely, apoptosis [2, 24, 25] and autophagy [26–48], also play an important role.

Mammalian autophagy is an intracellular self-digesting pathway responsible for the removal of long-lived proteins, damaged organelles, and malformed proteins during biosynthesis by lysosomes [49]. Autophagy is divided into three main types according to the different pathway by which cargo is delivered to the lysosome or vacuole: chaperone-mediated autophagy (CMA), microautophagy, and macroautophagy [50]. The last type can be divided into six principal steps: initiation, nucleation, elongation, closure, maturation, and degradation or extrusion [49]. Through these steps cytoplasmic materials, such as protein aggregates and organelles, are sequestered by the phagophore, a preautophagosomal membrane structure, which thereafter expands and encloses its cargo to form a double-membrane vesicle, the autophagosome [51]. By its fusion with a lysosome, it forms an autolysosome, in which the enclosed cargo is degraded by acid hydrolases into biologically active monomers such as amino acids that are subsequently reused to maintain cellular metabolic turnover and homeostasis [51].

Autophagy is involved in normal and diseased liver [52–55]. Studies on autophagy in liver tissue following warm/cold I-R remain controversial. Some show defective and/or decreased autophagy [27–30, 32, 34–37, 39–41, 45, 47]; in others hepatocellular autophagy is increased [26, 31, 33, 38, 42–44, 46, 48]. Whether autophagy protects from or promotes liver injury following warm and/or cold I-R remains to be elucidated.

## 2. Pathomechanisms of Warm and/or Cold Liver I-R Injury

Warm ischemia of the liver is due to oxygen deficiency caused by vascular occlusion of the liver during liver resection or hemorrhagic shock [2, 10]. Cold ischemia is observed during liver transplantation in which the graft is subjected to warm and cold ischemia followed by a warm reperfusion phase; the warm/cold ischemia sequence is due to vascular occlusion of the liver graft during its procurement from the donor and to graft storage in cold preservation solutions before liver transplantation [2, 10]. Graft implantation into the recipient represents the warm reperfusion phase; all these phases lead to I-R injury of the liver [2, 10]. Pathomechanisms associated with warm I-R seem to differ from those after cold I-R in liver transplantation [57]. Unlike warm liver I-R, which results in primary damage to the hepatocytes, cold I-R is mainly characterized by injury to the sinusoidal lining cells [58–60].

The introduction of oxygenated blood during reperfusion aggravates the ischemic insult, which itself is mainly characterized by cellular adenosine triphosphate (ATP) depletion and results in perturbation of the cellular energy-dependent metabolic and transport processes [1, 61]. Although the graft metabolism is reduced during hypothermia, with a prolonged time period in which the anoxic cells can retain essential metabolic functions, hypothermia may induce liver injury by dysfunction of the Na/K ATPase membrane pump [61, 62]. The resulting intracellular influx and accumulation of sodium and chloride lead to a perturbation of calcium homeostasis and to cell swelling [61, 62]. The inflammatory response to liver I-R involves neutrophils, cytokines, chemokines, complement, monocytes, and macrophages [63–67]. The reperfusion process consists of two phases: in the initial phase, activated resident macrophages of the liver, the Kupffer cells, induce oxidative stress mainly by reactive oxygen species (ROS) generation and, in the later phase, 6–24 hours following reperfusion, recruited neutrophils release inflammatory mediators which can cause direct tissue damage [1]. Kupffer cells play a central role in the pathophysiological mechanisms of liver I-R. Activated Kupffer cells release both ROS and cytokines, including tumor necrosis factor alpha (TNF*α*), Interleukin-1 (IL-1), and Interleukin-6 (IL-6), leading to granulocytes accumulation in the sinusoidal space and causing microcirculatory disturbances [68]. Accumulation of activated neutrophils through release of oxidants and proteases leads to hepatocyte damage. ROS stimulate endothelial cells to secrete platelet activating factor (PAF) [69]. Monocytes and Kupffer cells-derived ROS activate redox-sensitive transcription factors AP-1 and nuclear factor kappa-B (NF-*κ*B) in endothelial cells and hepatocytes [65]. Complement activation products activate Kupffer cells during the initial phase of liver injury and contribute to tissue inflammation as a membrane-attacking complex that stimulates the production of proinflammatory cytokines and chemotactic agents, which occur immediately after reperfusion [63]. Complement can also regulate the adaptative immunity [70, 71]. In fact, the inflammation occurring during liver reperfusion is predominantly an innate-immune-dominated response, which might induce I-R injury in both parenchymal and nonparenchymal cells,* in situ* and in liver transplants [66, 67, 72, 73]. However, the cold preservation injury of the graft causes a likewise strong adaptative immune response characterized by an early and massive T-cell influx into the ischemic liver graft [73–75]. The conventional T lymphocytes, CD4+ cells, accumulate in the liver within 1 hour after reperfusion preceding any neutrophil accumulation [76]. CD4+ cells are activated by various Kupffer cell-derived products and lead to hepatocytes and sinusoidal endothelial cells damage [76] and finally liver cells necrosis [22, 23, 77]. Apoptosis seems to be a relevant death mechanism during warm/cold liver I-R injury too [2, 24, 25] even if the studies are controversial on this issue [21, 24, 25]. Recently, the role of liver autophagy following warm and/or cold liver I-R has been highlighted [78].

## 3. Autophagy and Warm and/or Cold Liver I-R Injury

**Figure 1:**
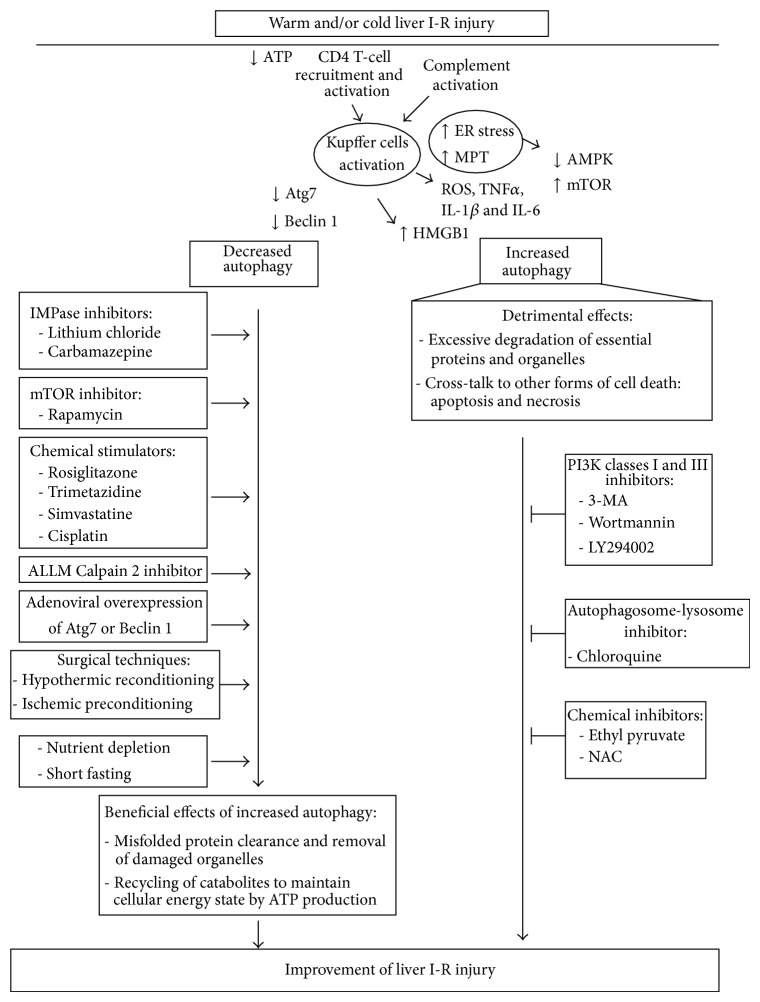
Pathomechanisms of warm and/or cold liver I-R injury and effects of modulation of autophagy. Pharmacological and/or surgical modulation of decreased or excessive autophagy during warm and/or cold liver I-R may improve liver injury. Arrow: stimulation; horizontal T: inhibition.

Beside necrosis [21–23] there are other modes of cell death, as apoptosis [2, 24, 25] and autophagy, that may occur simultaneously and or sequentially [26–48, 78] following warm and/or cold liver I-R. While some studies have shown a defective autophagy in hepatocytes following anoxia/reoxygenation and in liver tissue following I-R [27–30, 32, 34–37, 39–41, 45, 47], in others an increase of autophagy has been observed [26, 31, 33, 38, 42–44, 46, 48] (Table 1) with different effects on warm and/or cold liver I-R injury (Figure 1).

### 3.1. Protective Role of Autophagy against Warm and/or Cold Liver I-R Injury

#### 3.1.1. *In Vitro* and* In Vivo* Animal Studies

The mammalian orthologue of yeast autophagy-related gene 6 (Atg6), Beclin 1, has an important role in autophagosome formation as a component of multiprotein class III phosphatidyl inositol-3 kinase (PI3K) complex [79]. Beclin 1 is important for localization of autophagic proteins to preautophagosomal membrane structure during the nucleation step of autophagy [80]. Reduced Beclin 1 levels have been observed in hepatocytes during anoxia/reoxygenation in mice [37] and following 45 min warm liver I-R in rats [27]. Increased expression of the autophagic protein Beclin 1 by nutrient deprivation, by its pharmacologic induction, and by its adenoviral overexpression during anoxia/reoxygenation in mouse hepatocytes* in vitro* [37] and in rat livers following I-R injury* in vivo* [32] protects hepatocytes from cell death and reduces liver I-R injury [27, 32, 37].

Impaired liver autophagy seems to be mediated by calcium overload and consequent Calpain 1 and Calpain 2 activation which mediates the proteolytic clivage of the autophagic protein Beclin 1 and/or of Atg7 in anoxic hepatocytes [27] and in livers following total warm I-R [27, 45]. Defective autophagy may culminate in onset of mitochondrial permeability transition (MPT) and hepatocyte death after reoxygenation [27]. MPT results in either, necrosis by uncoupling of oxidative phosphorylation and apoptosis by releasing proapoptotic factors that are normally sequestered in the mitochondrial intermembrane space [77, 81]. MPT acts also as a molecular signal initiating the autophagic degradation of mitochondria, the mitophagy [82]. Impaired mitophagy following liver I-R fails to remove dysfunctional mitochondria, the mitochondria loaded with ROS undergo MPT, which in turn leads to uncoupling of oxidative phosphorylation, energetic failure, ATP depletion, and ultimately cell death [27, 82]. During liver I-R, Kupffer cells, neutrophils, and platelets are activated. Their activation results in a generation and release of ROS and in a cascade of inflammatory events including release of proinflammatory cytokines such as TNF*α*, IL-2, IL-6, IL-1, and high mobility group box 1 (HMGB1) protein [83]. HMGB1 is a DNA binding protein, which, when secreted actively by nonparenchymal liver cells (Kupffer cells and endothelial cells) and by neutrophils or when passively released by necrotic liver cells [84, 85], may induce an inflammatory signaling cascade [86]. HMGB1 acts as an alarmin, an alarm protein signal that initiates the inflammatory response resulting from liver I-R [87]. In normal rat liver, HMGB1 is mainly present in the nuclei of hepatocytes [87]. HMGB1 was released into the effluent collected from the infrahepatic vena cava during prolonged cold saline preservation of isolated rat liver grafts [88]. After cold liver graft preservation for 6 hours and transplantation in rats, serum levels of HMGB1 were increased in the early reperfusion phase [89]. Following 60 warm partial liver I-R in mice [86] and following 90 min warm partial liver I-R in rats [90], HMGB1 translocated from the nucleus to the cytoplasm of hepatocytes and was released into the blood circulation within 1 hour after reperfusion. During warm liver I-R, tissue levels of HMGB1 increase with its innate immune activation requiring toll-like receptor 4- (TLR4-) dependent signaling already 1 hour following reperfusion and continue to increase for up to 24 h later [87]. In human liver transplantation, the peak of HMGB1 serum levels was observed 10 min after reperfusion; thereafter, it started to decrease progressively within 1-2 hours [91].* In vitro*, nontoxic concentrations of Cisplatin, a platinating chemotherapeutic, can sequester HMGB1 inside the nucleus of hypoxic rat hepatocytes, can increase Beclin 1 expression, can modulate liver I-R-induced MAPK activation, and can induce autophagy [32]. These abilities of Cisplatin had beneficial effects on warm liver I-R injury and were also observed* in vivo* in mice [32]. In fact,* in vivo* administration of nontoxic concentrations of Cisplatin prevented HMGB1 release induced by 60 min partial warm liver I-R and reduced subsequent liver injury in mice [32]. Liver I-R alone increased Beclin 1 and Atg8/microtubule-associated protein 1 light chain 3 (LC3) expression; LC3 is also involved in the formation and expansion of the autophagosome; however, after Cisplatin administration, this increase was more pronounced and associated to mitophagy and finally proved to be protective against liver I-R injury [32].

The processing and degradation of LC3, from the unconjugated form microtubule-associated protein 1 light chain 1 (LC3-I) to the conjugated form microtubule-associated protein 1 light chain 2 (LC3-II), which remains associated to the autophagolysosome, indicate an increase in autophagy [49–51].

In rats, 60 min partial warm liver ischemia resulted in increased liver LC3-II. LC3-II is associated with the autophagosomal membrane allowing the closure of the autophagic vacuole and increases Atg5 expression which, when associated in a protein complex with Atg12 and Atg16, leads to autophagosome formation and finally to increased autophagy during reperfusion [42]. This liver I-R-induced LC3-II and Atg5 expression was more pronounced after chronic Lithium pretreatment of rats [42]. In fact, Lithium can induce autophagy by inhibiting inositol monophosphatase (IMPase) and leads to free inositol depletion which in turn may decrease myo-inositol-1,4,5-triphosphate (IP3) levels [92]. Induction of liver autophagy by chronic Lithium treatment before induction of 60 min partial warm ischemia was associated with reduced I-R liver injury, lower hepatic inflammatory cytokines levels, less liver neutrophil infiltration, and lower liver HMGB1 expression and serum HMGB1 levels [42].

It has been shown that the serine/threonine kinase Akt, also known as protein kinase B (PKB), plays a key role in cell survival and proliferation and may protect against liver I-R injury [93–96]. Hepatocytic anoxia/reoxygenation and 90 min partial warm liver I-R in mice resulted in a moderate but significant increase of hepatocellular levels of autophagy [38]. Hydrogen sulphide (H_2_S) pretreatment of mice exerted a protective effect in both hepatocytic anoxia/reoxygenation and 90 min partial warm liver I-R injuries through Akt1 activation [38]. However, H_2_S pretreatment suppressed the moderate but significant increase of liver autophagy levels occurring during hepatocyte reoxygenation and during warm liver reperfusion. In both experimental conditions, pretreatment with the autophagic inducer Rapamycin, which induces autophagy by binding with cochaperone immunophilin FKBP1 to specifically inhibit the mammalian target of Rapamycin (mTOR) complex 1, can reverse the autophagic inhibition of H_2_S and enhances its hepatoprotective effects [38].

Reducing ROS-induced hepatocellular necrosis seems to be another protective role of autophagy in 90 min partial warm liver ischemia injury in rats [43]. Following 90 min partial warm liver ischemia without reperfusion, both increased levels of LC3-II and increased number of autophagosomes were observed [43]. These increases were associated with generation of mitochondrial ROS and liver injury [43]. When autophagy was inhibited by the autophagy inhibitor Chloroquine, the increase of mitochondrial oxidative stress, of ROS production, and of mitochondrial damage was even more pronounced [43]. The consequent accumulation of damaged mitochondria, which are normally sequestered and degraded through autophagy, leads to an enhanced ROS production with a subsequent acceleration of ischemia-induced liver injury and an increase of hepatocellular necrosis [97].

Decreased autophagy is a physiological consequence of aging [98].* In vitro* hepatocyte I-R and* in vivo* warm liver I-R injury associated with autophagy seems to be age-dependent [28, 34].* In vitro* old mice hepatocytes subjected to 2 hours of hypoxia followed by 12 hours of reoxygenation and* in vivo* 20 min total warm liver ischemia followed by 40 min reperfusion showed an increase in Calpain 2 activity, which hydrolyzed Atg4B and led to impaired autophagosome formation, impaired autophagic flux and mitophagy, and promoted the onset of the MPT and subsequent cell death [34]. The activation of peroxisome proliferator-activated receptor gamma (PPAR*γ*), which belongs to the hormone nuclear receptor superfamily, is downregulated during liver ischemia aggravating liver injury [99]. PPAR*γ* activation was lost in old mice after 30 min partial warm liver ischemia [28]. This suppression of PPAR*γ* activation was accompanied by a reduced liver autophagy, which was more important in correlation with the duration of ischemia. Pretreatment of mice with the autophagy inducer Rosiglitazone, activated PPRA*γ* and increased liver autophagy after warm ischemia [28].

Steatotic livers have an increased risk of postoperative complications after liver resection [100] and liver transplantation [101]. They are particularly susceptible to mitochondrial alterations after storage in cold preservation solutions for transplantation [102, 103]. Steatosis may provide a substrate that promotes not only oxidative stress but indirectly also oxidant injury by decreasing the autophagic function [104, 105]. An impaired autophagy was observed during cold I-R of steatotic rat livers [40, 41]. Pretreatment with some autophagic stimulators, as well as Trimetazidine and Simvastatine, increased liver autophagy and improved I-R injuries of steatotic rat livers submitted to cold I-R [40, 41].

The use of hypothermic reconditioning (HR), a surgical technique consisting of temporary hypothermic oxygenation of the grafts by using an oxygenated machine perfusion or gaseous oxygen persufflation, may improve graft function and viability [106, 107]. HR of steatotic livers by insufflation of gaseous oxygen via the cava vein during the last 90 minutes of cold preservation of the graft has limited mitochondrial dysfunction and restored basal rates of hepatocellular autophagy in rats [30]. The beneficial effects of HR on liver grafts have also been shown in transplantation of nonsteatotic pig livers, where hepatocellular autophagy was preserved, mitigating the activation of innate immunity and leading to an improved survival of recipients [36].

#### 3.1.2. *In Vitro* and* In Vivo* Human Studies

In primary isolated human hepatocytes, autophagy is a cell survival mechanism during oxidative stress [39]. Isolated primary human hepatocytes, which were exposed* ex vivo* to hypoxia and hypoxia-reoxygenation, showed an increase of autophagy within the mitochondria [39]. Inhibition of autophagy by 3-methyladenine (3-MA) in these stressed hepatocytes resulted in the lowering of MPT and onset of cell death by apoptosis [39]. During warm and cold liver I-R, ROS are responsible for lipid peroxidation, protease activation, cytokine release, adhesion molecule expression, microcirculatory failure, and finally apoptosis and necrosis [63]. However, ROS are also critical mediators of autophagy [108] and, during hypoxia-reoxygenation of primary human hepatocytes, inhibition of their production by N-acetylcysteine (NAC), Rotenone, and Diphenyliodonium suppressed autophagy and led to reduced levels of apoptosis and necrosis [39]. Thus, ROS seem to be key mediators of autophagy during oxidative stress and, depending on either the absolute level of intracellular ROS, the type of ROS subspecies generated, or the duration of ROS generation, they may be critical factors in determining cell death by apoptosis or necrosis [22, 23, 39].

Ischemic preconditioning (IP) of the liver is a surgical procedure consisting of a short period of liver ischemia (10 min) followed by reperfusion (10 min). Then the prolonged period of ischemia by clamping the hepatic artery and the portal vein on the hepatic pedicule (Pringle maneuver) is better supported by the liver [109]. After promising results in animal models, IP was efficiently used in clinical studies [110, 111]. However, its benefit to protect the liver from I-R injury in liver resection and transplantation in humans remains controversial [112–115]. A recent meta-analysis of IP for liver resection in patients with and without chronic liver diseases failed to find a significant benefit of IP in liver resection [115]. Also, in liver transplantation, there were no clear benefits of IP [112, 113]. Hepatosteatosis and vascular injury induced by chemotherapy can reduce tolerance of the liver to reperfusion injury and increase the risk of subsequent liver failure [116, 117]. In steatotic human livers formerly treated by chemotherapy, the use of IP before prolonged ischemia required by liver resection resulted in limited hepatocyte necrosis and was associated with an activation of liver autophagy [29]. The beneficial effects of IP on liver I-R injury seem to be a consequence of autophagy onset leading to preserved ATP levels and avoiding hepatocellular necrosis by delaying proapoptotic effects [29]. In liver transplantation IP of steatotic grafts (preconditioning in the donor before graft removal) induced autophagy, limited necrosis in human recipients, and decreased the incidence of rejection episodes [35]. In the ischemic preconditioned steatotic graft, a cellular increase of Beclin 1 and LC3 was observed, compared with non-IP steatotic liver grafts [35]. In addition, there was an inverse correlation between the number of LC3-positive cells and the necrotic index in IP steatotic liver grafts [35]. IP decreased the incidence of both acute and chronic liver rejection in recipients of steatotic grafts compared to recipients of non-IP steatotic grafts [35].

Overall, these studies showed that restoration or enhancement of autophagy may improve liver I-R injury by providing cells with the energy derived from lysosomal degradation of cellular materials [118].

### 3.2. Detrimental Role of Autophagy in Warm and/or Cold Liver I-R Injury

#### 3.2.1. *In Vitro* and* In Vivo* Animal Studies

Several studies have shown increased levels of autophagy in hepatocytes following anoxia/reoxygenation and in livers following warm or cold/warm I-R [26, 31, 33, 38, 42–44, 46, 48]. These increased levels of autophagy appeared, either detrimental [26], probably protective [31], or both protective in the early phase of reperfusion and detrimental in the late phase of liver reperfusion [44]. Furthermore, when pharmacologically modulated, inhibited [33, 46, 48] or stimulated [38, 42, 43] autophagy improved warm and/or cold liver I-R injury in both cases.

Increased autophagy was observed following partial 120 min warm liver ischemia in rats 6 hours after reperfusion [31]. The hepatocytes of the ischemic liver lobes, 6 hours after reperfusion, had dense bodies and various autophagosomes as well as oval and rounded mitochondria [31]. The number of hepatocytes with punctate LC3 staining in the cytoplasm was markedly increased in ischemic compared to nonischemic liver lobes [31]. During orthotopic LT in rat, autophagosomes/autolysosomes were observed in graft hepatocytes in both cold preservation and reperfusion phases [26]. Induction of autophagy was more pronounced in graft hepatocytes after 30 to 60 min of warm reperfusion than in hepatocytes after cold preservation [26]. Abundant autophagosomes/autolysosomes were associated with dying hepatocytes within 2 hours of warm reperfusion [26]. Warm reperfusion phase may facilitate autophagosome formation in hepatocytes under ATP exhaustion as a stress response [26]. About 15 minutes after the start of warm reperfusion, small masses of hepatocytes with abundant autophagosomes/autolysosomes frequently dissociated from the hepatic cords and were extruded into the sinusoidal lumen [33]. Occlusion of the sinusoidal stream contributed to a massive necrosis of hepatocytes within 2 hours and led to liver dysfunction [33]. The hepatocytes containing numerous vacuolar/lysosomal structures often underwent degeneration and were phagocytosed by Kupffer cells late in the reperfusion phase [26]. The inhibition of autophagosome formation and maturation by adding the autophagic inhibitor Wortmannin and LY294002, a specific inhibitor of PI3K/Akt kinase pathway, to the cold preservation solution attenuated liver dysfunction and recipient mortality rates [33].

The beneficial effects of Rapamycin by reversing the autophagic inhibition of H_2_S during hepatocytic anoxia/reoxygenation and 90 min partial warm liver I-R in mice [38] are in contrast with the detrimental effects of Rapamycin on reperfused livers following partial warm 60 min liver ischemia in mice shown in a recent study [48]. In fact the increased levels of autophagy induced by warm reperfusion were even higher after Rapamycin pretreatment and this excessive activation of autophagy aggravated liver I-R injury [48]. Furthermore, in the same study, Rapamycin reversed the beneficial effects of Melatonin administration, a lipophilic indole secreted by pineal and nonpineal cells, which seems to protect against liver I-R injury by inhibiting oxidative stress and by improving both mitochondrial respiration and ATP synthesis after cold storage of the liver [119, 120]. In this study, Melatonin downregulated autophagy via activation of mTOR signaling and resulted in improvement of liver I-R injury [48].

In contrast, in moderate and advanced steatotic cold-stored and warm-reperfused livers in rats, in which autophagy was impaired [40], Melatonin associated with Trimetazidine, induced liver autophagy, and improved liver injury [41]. When adding Simvastatine, a statin possessing vasoprotective properties, to the cold-storage solution, the bioavailability of the vasoprotector NO was maintained and led to autophagy induction [40]. Simvastatin treatment prevented hepatic endothelial dysfunction not only in steatotic [40] but also in nonsteatotic [121] livers and resulted in improvement of liver injury.

An increased level of autophagy was observed also in rat liver following 60 and 90 min partial warm liver I-R [44]. LC3-II protein was increased at 6 hours after liver reperfusion; this increase was more pronounced when rats were pretreated with the antimalaria drug Chloroquine [44]. Chloroquine seems to have a dual role in warm liver I-R; in fact, it improved liver injury in the early phase of reperfusion by reducing inflammatory cytokines, as well as IL-6, IL-1, and TNF*α*; it diminished HMGB1 release, and it modified I-R-induced MAP kinase activation [44]. In the late phase of reperfusion, however, Chloroquine inhibited autophagy and induced apoptosis aggravating liver I-R injuries [44]. By contrast, in another study, levels of autophagy were decreased earlier in hepatocytic anoxia/reoxygenation and at 1 and 4 hours following partial warm 60 min liver I-R* in vivo* in rats [47] and, after Chloroquine treatment, liver I-R injuries were aggravated at these time points studied [47].

HMGB1 has also an important functional role in cross-regulating apoptosis and autophagy [80]. Actively secreted by nonparenchymal liver cells, Kupffer, and sinusoidal endothelial cells and/or passively released by necrotic liver cells [84, 85], the stress sensor with redox activity HMGB1 acts as protein signal that initiates the inflammatory response resulting from liver I-R [87]. For HMGB1 translocation from nuclei to cytoplasm and for enhanced autophagy, ROS signals are required [122]. Following 45 min warm partial liver I-R injury in mice, HMGB1 translocated from the nucleus to the cytoplasm of hepatocytes, competitively combined with Beclin 1 and promoted the levels of autophagy through representing the site of the antiapoptotic Bcl-2 protein, which normally maintains the inactive status of autophagy, and caused warm liver I-R injury in rat [46].

Ethyl-Pyruvate, a lipophilic ester derived from the endogenous metabolite pyruvate, seems to improve liver injury by inhibiting the intrinsic pathway of apoptosis and autophagy [46]. Ethyl-Pyruvate might decrease separately both apoptosis through the downregulation of the HMGB1/TLR4/NF-*κ*B axis and autophagy through competitive interaction with Beclin 1 [46]. In fact, following 45 min warm partial liver I-R injury in mice, stressed hepatocytes released HMGB1 [46]. When animals were treated with Ethyl-Pyruvate, liver injury, apoptosis, and necrosis were decreased as a result of HMGB1 downregulation and autophagy was inhibited through the competitive interaction of HMGB1 with Beclin 1 [46].

Nevertheless, a recent study suggested that HMGB1 is not required for ATP production, cellular respiration, mitochondrial architecture, or autophagy in liver and heart [123]. In mice with conditional HMGB1 deletion, mitochondrial function and liver and heart autophagy were not affected [123]. Other studies again outline the key role of HMGB1 in mitochondrial quality control and autophagy [124–126]. In particular, an aggravation of liver I-R injury after HMGB1 depletion has been reported. In fact, genetic deletion of HMGB1 from hepatocytes resulted in enhanced inflammatory signaling, led to nuclear instability with increased DNA damage and histone release, led to mitochondria damage by exhausting nicotinamide adenine (NAD) and ATP stores, exhibited increased ROS production, and finally increased cell death [126]. In mice, fasting for one day protected from 60 min warm liver I-R injury via Sirt1-dependent downregulation of circulating HMGB1 [127]. The reduced levels of circulating HGMB1 damped the activation and self-propagation of Kupffer cells and hence protected from liver I-R [127].

### 3.3. Protective or Detrimental Role of Autophagy against Warm and/or Cold Liver I-R Injury: Various Drugs and Different Methods for Monitoring Autophagy in Different Animal and Human Experimental Liver Warm and/or Cold I-R Models

How pharmacological and surgical modulation of liver autophagy could protect from or promote liver injury following I-R remains to be clarified, as the studies on this subject report either downregulated or excessive levels of liver autophagy. At this point, it is important to mention that the methods used for measuring autophagy were mainly steady state methods, the drugs used to modulate liver autophagy were not entirely specific for inhibition or stimulation of autophagy, and the animal and human experimental liver I-R models differed considerably. In order to study the effects of autophagy modulation on warm and/or cold liver I-R injury both animal experimental liver I-R models and human experimental I-R models are used. The common length of partial warm ischemia in rodent models was usually 30, 45, 60 or 90 min [128]. In agreement with this, as shown in Table 2, in most of studies, the role of autophagy on liver I-R injury in rodents has been evaluated by using 60 min partial lobar (70%) liver warm ischemia model [28, 32, 37, 42, 44, 47, 48]. The model of partial lobar (70%) liver ischemia includes interruption of blood flow to the left lateral and median liver lobes leaving the right lobe for decompression [129]. As animal studies overall mimic real clinic conditions in which liver surgery and liver transplantation are performed in humans, they permit to draw conclusions with certain relevance for the human physiopathology.

#### 3.3.1. Methods for Monitoring Autophagy

Accumulation of autophagosomes may be due to both the induction of autophagy and the blockage of a late step of the autophagy process, including impaired autophagosome-lysosome fusion and compromised lysosomal activity [130]. Induction of autophagy, assessed by steady state methods, does not allow a determination of whether the autophagic process goes to completion [56]. Incomplete autophagy, which would lead to the accumulation of autophagosomes, may contribute to cellular and organ dysfunction, whereas complete autophagy will generally exert a cytoprotective effect [56]. As steady state methods evaluate autophagy only at a certain time point [56], they may not reflect properly the autophagic activity [56]. Actually, Table 2 shows that most studies on warm and/or cold liver I-R [26, 28–33, 35–42, 44, 46] used steady state methods: electron microscopy, Atg8/LC3 western blotting and ubiquitin-like protein conjugation systems, fluorescence microscopy for monitoring phagophore, and autophagosome formation.

*Electron Microscopy.* The autophagosome is a transient organelle existing for less than 10 min before fusing with the lysosome, resulting in the appearance of autophagolysosomes at various stages of degradation [131]. Electron microscopy can visualize early-stage autophagosomes but is less sensitive for the visualization of late-stage autophagosomes [132]. So the isolated approach with electron microscopy is not sufficient to evaluate autophagy levels [56].

*LC3 Western Blotting.* LC3-II is present in most of the autophagic steps and reliably associated with phagophores, sealed autophagosomes and mature autophagosomes/autolysosomes [133]. It is widely used to monitor autophagy. Immunoblot analysis detects the conversion of LC3-I to LC3-II; the amount of LC3-II is clearly correlated with the number of autophagosomes [134]. However, LC3-II itself is degraded by autophagy, and the amount of LC3-II at a certain time point does not necessarily indicate autophagic flux. Simple comparison of LC3-I and LC3-II or summation of LC3-II and LC3-II for ratio determination may not be appropriate to correctly evaluate autophagy [134]. An increased number of autophagosomes can occur despite later steps of autophagy being blocked; the quantification of LC3-II [134] before and after the inhibition of autophagosome-lysosome fusion by using lysosomal inhibitors may indicate more accurately the autophagic flux [134]. Chloroquine and hydroxychloroquine increase the pH of the lysosome; Bafilomycin A1 inhibits the lysosomal Na^+^H^+^ ATPase; in this way, they prevent the activity of lysosomal acid proteases and cause autophagosomes to accumulate. Similar effects are induced by treatment with specific inhibitors of lysosomal proteases, such as Pepstatin A and/or E64d [134]. In this case, the real autophagic flux is represented by the different amounts of LC3-II in the samples in the absence or presence of lysosomal proteases inhibitors. LC3-II levels proportionally increase in treated versus untreated samples [134].

*Ubiquitin-Like Protein Conjugation Systems.* The p62 (SQSTM1/sequestosome 1) is a ubiquitin-binding scaffold protein that can bind LC3 [135]. This protein accumulates when autophagy is inhibited and decreases when autophagy is induced [136]. In some studies on warm or cold liver I-R injury in rats, autophagy was monitored by degradation of p62 using Western blot method [41, 42, 44]. However, p62 is regulated at the transcriptional level by oxidative stress and by Ras oncogene and also feeds back to regulate NF-*κ*B activity [136]. As p62 levels may be changed independently from autophagy; additional methods to validate changes in protein aggregate turnover by autophagy are necessary [136–138]. 

*Fluorescence Microscopy.* The fluorescent-based method with the green fluorescent protein- (GFP-) LC3 counting the GFP-LC3 puncta uses the fact that, after autophagy induction, LC3B becomes part of the newly formed autophagosomes and that GFP-LC3 changes its cellular localization from a diffuse cytosolic pattern to a punctate pattern. Once again, as steady state measurement, this method is not sufficient to measure autophagy, when used as an isolated approach [56].

Autophagy is a dynamic process of bulk degradation of cellular proteins and organelles in lysosomes [139]. Autophagic substrates need to be monitored to verify that they have reached these organelles and eventually degraded [56]. Evaluation of the autophagic flux, a complete process of autophagy including the delivery of cargo to lysosomes, via its fusion with autophagosomes or amphisomes and its subsequent breakdown and recycling, [56] is important to determine whether drugs and/or surgical techniques truly affect autophagy. As shown in Table 2, only a few studies have monitored the autophagic flux in order to evaluate the extent of autophagy [27, 34, 43, 45, 47, 48].

#### 3.3.2. Side Effects of Chemical Autophagy Stimulators or Inhibitors

The chemical stimulators and inhibitors of autophagy used actually are not specific and may have a series of additional effects on liver I-R apart from their action on autophagy.

*(1) Chemical Stimulators of Autophagy.* Inducing, increasing, or restoring basal autophagic activity in certain cell types as the hepatocytes, following warm and/or cold liver I-R, might be of therapeutic benefit. As shown in Table 2, the modulation of autophagy by some stimulators protected against liver I-R injury [27, 28, 32, 34, 37, 38, 41, 42, 45]. However, the unspecificity of the used drugs renders the interpretation of the results difficult.

Rapamycin, an autophagy inducer by inhibiting the mTOR pathway, plays a central role in several important cellular processes other than autophagy [140]. Interfering with the translation of HIF-1*α*, it has an antiangiogenic effect, and inhibiting the phosphorylation of BAD by S6 K1 Rapamycin may promote apoptosis [140]. So its protective effects in 90 min warm liver I-R injury in mice may be partly due to Akt1 activation and decreased apoptosis by H_2_S associated copretreatment [38]. Similarly Rosiglitazone, another autophagy stimulator, has shown additional protective effects (increase of ATP levels and the inhibition of Caspase-3 activation) in 30, 60, and 90 min liver I-R injury in old mice that cannot be accounted to the increase of autophagy [28].

The IMPase inhibitors, Lithium chloride and Carbamazepine, can induce autophagy [92]. They have also shown additional protective effects against liver I-R injury other than autophagy induction [42, 45]. Lithium chloride showed multiple additional effects, the decrease of HMGB1 and proinflammatory cytokines levels, the modulation of MAPK activation, and the inhibition of Caspase-3 and Caspase-7 activation [42]. The second, Carbamazepine, suppressed both calcium overloading and uncontrolled Calpain activation [45]. In a murine model of 60 min liver I-R Cisplatin treatment increased liver autophagy and protected against I-R injury [32]. Although the beneficial effects of Cisplatin are not only the result of autophagy stimulation but also of decreased HMGB1 and proinflammatory cytokines levels and of the modulation of MAPK activation [32].

In steatotic rat livers that were preserved in cold solution for 24 hours, autophagy was decreased [41]. A close relationship between 5′ AMP-activated protein kinase (AMPK) activation and endoplasmic reticulum (ER) stress and autophagy has been observed in these livers [41]. The addition of a Melatonin and Trimetazidine cocktail to the cold preservation solution improved steatotic liver graft preservation through AMPK activation, which in turn reduced ER stress and increased autophagy [41]. However, these beneficial effects of the Melatonin and Trimetazidine cocktail may be due in part to a reduction of apoptotic liver cells mainly observed in periportal and midzonal areas of the liver [41].

In human liver surgery and transplantation, the improvement of clinical outcomes by the use of IP of the liver was associated to higher levels of autophagy. However, the decrease of ER stress [35] and the increase of ATP and Beclin 1 levels observed after IP of the liver may have been responsible too for the beneficial effects of this surgical strategy [29].

*(2) Chemical Inhibitors of Autophagy.* Similar to autophagy stimulators, autophagy inhibitors are not specific [33, 39, 43, 44] and additional effects of these drugs may play an important role [43, 44, 46–48].

Autophagy inhibition by 3-MA in human hypoxemic/reoxygenated hepatocytes resulted in MPT lowering and onset of apoptosis [39]. Autophagy inhibition by LY294002 and Wortmannin during liver transplantation in rats reduced liver graft dysfunction and mortality rate of transplanted animals [33].

The PI3K inhibitors, 3-MA, LY294002, and Wortmannin, typically block Class III PI3Ks, which act as downstream of the negative regulatory Class I kinase. However, their inhibitory action on autophagy differs [141]. In fact 3-MA may promote or suppress autophagic flux [142], whereas Wortmannin seems to have inhibitory effects opposite to those of 3-MA. It has persistent effects on class III PI3K, an autophagy activator, but also it has transient effects on class I PI3K, which is an autophagy inhibitor [142]. The autophagic inhibitor LY294002 plays also a dual role in the regulation of autophagy, as it may activate autophagy by inhibiting the class I PI3K [143] and as it modulates calcium overloading, Calpain activation, and MPT, all implicated in the development of warm liver I-R injury [77, 81].

Also Melatonin, Ethyl-Pyruvate, and Chloroquine, all three autophagy inhibitors, present additional protective effects against liver I-R injury. Melatonin in fact may decrease liver apoptosis [48], Ethyl-Pyruvate may inhibit HMGB1/TLR4/NF-*κ*B axis inducing apoptosis [46] and Chloroquine, which has protective effects in the early reperfusion phase after 60 and 90 min warm liver ischemia, may modulate MAPK activation [44].

Autophagy inhibition by Chloroquine pretreatment increased mitochondrial oxidative stress and hepatocellular necrosis following 90 min warm liver I-R in rats [43]. Antioxidant NAC pretreatment again diminished the ischemia-induced liver injury of these rats, which received also Chloroquine treatment. However, the beneficial effects against liver injury by NAC seem to be due also to decreased mitochondrial ROS-inducing necrosis [43].

## 4. Conclusion

The pathogenesis of warm and/or cold liver I-R injury represents a complex interplay between necrosis, apoptosis, and autophagy. It seems that stimulation of autophagy plays a more important role during liver reperfusion than ischemia. Depending on the context, induction or impairment of autophagy during warm and/or cold liver I-R can be protective or detrimental for liver cells. Stimulation of impaired autophagy following warm and/or cold I-R may promote hepatocyte survival by degradation of intracellular contents to maintain ATP production and removal of damaged organelles and protein aggregates. Excessive and long-term upregulation of autophagy, as it occurs during severe ischemic insult of the liver, may lead to destruction of essential proteins and organelles resulting in hepatocellular apoptosis and necrosis.

However, the results of the studies on autophagy during warm and/or cold liver I-R remain discordant. This may be due to several factors, namely, the lack of drugs which exert specific and exclusive autophagic stimulation or inhibition, the different experimental liver I-R models used, and the different methods of autophagy evaluation. So how pharmacological and/or surgical modulation of liver autophagy could protect from or promote liver injury following warm and/or cold I-R remains to be clarified. Large animal studies on liver I-R, also at a genetic level with knockout models, which provide a very specific targeted disruption of a particular autophagic protein and will therefore be more informative than the use of not entirely specific chemical stimulators or inhibitors of the same autophagic protein, are needed. Last but not least the methods for monitoring autophagy should preferentially measure the autophagic flux.

In any way, the autophagic cell response to warm and/or cold liver I-R may provide an additional time to the cell death processes, delaying apoptosis and necrosis, and thus ultimately increasing the possibility for novel therapeutic intervention to diminish the extent of warm/cold liver I-R injury.

## Figures and Tables

**Table 1:** Autophagy and warm and/or cold liver I-R injury: *in vitro* and *in vivo* animal and human studies.

Species	Preexisting liver disease	Experimental liver I-R model	Effects on autophagy	Modulation of autophagy by inhibitors	Modulation of autophagy by stimulators	Effects on I-R injury	Authors and references
Rat	No	Partial warm 60 min	Decreased	(a) Hemin + Chloroquine (b) Hemin + Wortmannin		Detrimental Detrimental	Yun et al. 2014 [47]

Mice	No	Partial warm 60 min	Increased	Melatonin		Protective	Kang et al. 2014 [48]

Mice	No	Partial warm 45 min	Increased	Ethyl pyruvate		Protective	Shen et al. 2013 [46]

Mice	No	Total warm 45 min	Decreased		Carbamazepine	Protective	Kim et al. 2013 [45]

Rat	No	Partial warm 60 and 90 min	Increased	Chloroquine		Protective (early reperfusion phase) Detrimental (late reperfusion phase)	Fang et al. 2013 [44]

Rat	No	Partial warm 90 min	Increased	Chloroquine Chloroquine + NAC		Detrimental Protective	Sun et al. 2013 [43]

Rat	No	Partial warm 60 min	Increased		Lithium	Protective	Liu et al. 2013 [42]

Rat	Steatosis	Cold ischemia 24 h	Decreased		Melatonin + Trimetazidine	Protective	Zaouali et al. 2013 [41]

Rat	Steatosis	Cold ischemia 16 h/30 min warm reperfusion *in situ *	Decreased			Detrimental	Gracia-Sancho et al. 2013 [40]

Human	No	Hypoxia/reoxygenation of hepatocytes	Decreased	3-MA		Protective	Bhogal et al. 2012 [39]

Mice	No	(a) Anoxia/reoxygenation of hepatocytes	Increased		Rapamycin	Protective	Wang et al. 2012 [38]
		(b) Partial warm 90 min	Increased		Rapamycin	Protective	

Calcium/ Calmodulin- kinase IV KO mice	No	Partial warm 60 min	Decreased		Rapamycin	Protective	Evankovich et al. 2012 [37]

Human	No	Liver transplantation	Decreased		Ischemic preconditioning	Protective	Degli Esposti et al. 2011 [35]

Old mice	No	Total warm 20 min	Decreased		(a) ALLM Calpain 2 inhibitor (b) Adenoviral overexpression of Atg4B and Beclin 1	Protective	Wang et al. 2011 [34]

Pig	No	Liver transplantation	Decreased		Hypothermic reconditioning by gaseous oxygen	Protective	Minor et al. 2011 [36]

Rat	Steatosis	Cold ischemia 20 h	Decreased		Hypothermic reconditioning by gaseous oxygen	Protective	Minor et al. 2009 [30]

Mice	No	Partial warm 60 min	Decreased		Cisplatin	Protective	Cardinal et al. 2009 [32]

Rat	No	Liver transplantation	Increased	(a) Wortmannin (b) LY294002		Protective	Gotoh et al. 2009 [33]

Human	Postchemotherapy steatosis	Total Warm	Decreased		Ischemic preconditioning	Protective	Domart et al. 2009 [29]

Old mice	No	Partial warm 30, 60, and 90 min	Decreased		Rosiglitazone	Protective?	Shin et al. 2008 [28]

Rat	No	(a) Total warm 45 min	Decreased			Protective	Kim et al. 2008 [27]
		(b) Anoxia/reoxygenation of hepatocytes	Decreased		(a) Nutrient depletion		
					(b) Adenoviral overexpression of Atg7 and Beclin 1		
					(c) ALLM Calpain 2 inhibitor		

Rat	No	Partial warm 120 min	Increased			Protective?	Cursio et al. 2010 [31]

Rat	No	Liver transplantation	Increased			Detrimental	Lu et al. 2005 [26]

**Table 2:** Autophagy and warm and/or cold liver I-R injury. Methods for monitoring autophagy and additional beneficial effects of drugs and surgical techniques on liver I-R injury other than modulation of autophagy.

Authors and references	Monitoring autophagy by flux measurements [56]	Monitoring phagophore and autophagosome formation by steady state methods [56]	Additional beneficial effects of drugs and surgical techniques on liver I-R injury other than modulation of autophagy
Yun et al. 2014 [47]	(+)		HO-1 induction and Calpain 2 inhibition by Hemin

Kang et al. 2014 [48]	(+)		Decrease of apoptosis

Shen et al. 2013 [46]		(+)	Inhibits HMGB1/TLR4/NF-κB axis inducing apoptosis

Kim et al. 2013 [45]	(+)		Suppression of calcium overloading
			Suppression of uncontrolled Calpain activation

Fang et al. 2013 [44]		(+)	Decrease of HMGB1 and proinflammatory cytokines levels
			Modulation of MAPK activation

Sun et al. 2013 [43]	(+)		Decreased mitochondrial ROS-inducing necrosis by NAC

Liu et al. 2013 [42]		(+)	Modulation of MAPK activation
			Inhibition of Caspase-3 and -7 activation
			Decrease of HMGB1 and proinflammatory cytokines levels

Zaouali et al. 2013 [41]		(+)	Decrease of apoptosis

Gracia-Sancho et al. 2013 [40]		(+)	Decrease of the oxidative stress and Caspase-3 activation by Simvastatine


Bhogal et al. 2012 [39]		(+)	

Wang et al. 2012 [38]		(+)	Akt1 activation and decrease of apoptosis by H_2_S

Evankovich et al. 2012 [37]		(+)	

Degli Esposti et al. 2011 [35]		(+)	

Wang et al. 2011 [34]	(+)		

Minor et al. 2011 [36]		(+)	Decrease of HMGB1 and IFN beta levels

Minor et al. 2009 [30]		(+)	ROS decrease
			ATP increase

Cardinal et al. 2009 [32]		(+)	Decrease of HMGB1 and proinflammatory cytokines levels
			Modulation of MAPK activation

Gotoh et al. 2009 [33]		(+)	

Domart et al. 2009 [29]		(+)	Bcl-2 increase

Shin et al. 2008 [28]		(+)	ATP increase
			Inhibition of Caspase-3 activation

Kim et al. 2008 [27]	(+)		

Cursio et al. 2010 [31]		(+)	

Lu et al. 2005 [26]		(+)	
